# Investigation of Field‐Based Molecular Evidence of Natural Transovarial Transmission of *Babesia ovis* in *Rhipicephalus bursa* and *Rhipicephalus turanicus* Ticks

**DOI:** 10.1155/tswj/2474599

**Published:** 2025-12-17

**Authors:** Mehmet Can Ulucesme, Munir Aktas

**Affiliations:** ^1^ Department of Parasitology, Faculty of Veterinary Medicine, University of Fırat, Elazig, Türkiye, firat.edu.tr

**Keywords:** *Babesia ovis*, natural transovarial transmission, *Rhipicephalus bursa*, *Rhipicephalus turanicus*, Türkiye

## Abstract

*Babesia ovis* is a tick‐borne protozoan parasite that poses a significant threat to sheep production, particularly in endemic regions such as Türkiye. It is known to be transmitted by ixodid ticks through both transstadial and transovarial routes. This study was aimed at investigating the natural transovarial transmission potential of *B. ovis* in *Rhipicephalus bursa* and *Rhipicephalus turanicus* ticks under field conditions. A total of 751 adult ticks were collected from 84 small ruminants (41 sheep and 43 goats) in Elazığ Province, Türkiye. Following species identification, 85 fully engorged female *R. bursa* (*n* = 45) and *R. turanicus* (*n* = 40) were incubated under controlled conditions to allow oviposition. DNA was extracted from the female carcasses and their corresponding larval pools and analyzed using nested PCR targeting the *18S rRNA* gene of *B. ovis*. The results revealed that *B. ovis* DNA was detected in 19.04% (8/42) of *R. bursa* females and in 7.89% (3/38) of *R. turanicus* females. Importantly, all larval pools derived from *B. ovis*‐positive *R. bursa* females also tested positive, indicating natural transovarial transmission. In contrast, none of the larval pools from *R. turanicus* tested positive, despite the presence of *B. ovis* in the female carcasses. These findings suggest that *R. bursa* may serve as a competent natural vector and reservoir for *B. ovis*, whereas *R. turanicus* seems to possess less ability for vertical transmission of the parasite.

## 1. Introduction

The transmission of pathogens by ticks occurs through various mechanisms. The principal modes include horizontal transmission (between tick and host), transstadially (from one developmental stage of the tick to the next), and transovarially (from an infected female to its offspring) [1, 2]. Certain protozoan pathogens, such as *Theileria* species, are transmitted solely via transstadial passage in vector ticks [3], whereas *Babesia* species belonging to the *Babesia* sensu stricto group, including *Babesia ovis*, can be transmitted through all of the aforementioned routes [2]. Importantly, for a tick species to be considered a competent vector, it should demonstrate not only horizontal transmission but also at least one additional route among these modes [4, 5]. Furthermore, if a tick species is capable of both transstadial and transovarial transmission, it is not only a vector but may also serve as a reservoir host for the pathogen [5].

*B. ovis* is widely recognized as one of the most economically important protozoan parasites affecting small ruminants, particularly sheep, in many regions across the globe [6–11]. This tick‐borne hemoprotozoan is responsible for ovine babesiosis, a disease that poses a significant threat to sheep production systems, especially in endemic areas. In Türkiye, *B. ovis* infections have been reported in nearly all geographical regions, frequently leading to outbreaks associated with high morbidity and mortality rates among affected flocks [12]. Affected animals exhibit characteristic signs including high fever, progressive anemia, jaundice, and hemoglobinuria. In the absence of timely diagnosis and appropriate treatment, the infection can result in substantial mortality, particularly in young or immunocompromised animals [13, 14]. The life cycle of *B. ovis* is complex and involves both a vertebrate host (sheep) and an invertebrate vector (ixodid ticks). The parasite undergoes asexual reproduction (merogony) in the vertebrate host′s red blood cells, leading to clinical disease, while sexual reproduction (gamogony) and sporogony occur within the tick vector following ingestion of infected blood during feeding [15, 16]. This dual‐host life cycle not only facilitates the persistence and transmission of the parasite in nature but also complicates control strategies.

The geographical distribution of *B. ovis* is closely associated with several tick vectors, primarily *Rhipicephalus bursa*, as well as other species such as *Rhipicephalus turanicus*, *Rhipicephalus sanguineus*, *Rhipicephalus evertsi*, and *Hyalomma excavatum* [7, 17–20]. Among these, *R. bursa* has been experimentally confirmed as a competent vector capable of supporting the transovarial transmission of the parasite [14, 21, 22]. Additionally, *R. turanicus* has been reported as a potential vector for the transmission of *B. ovis* in Iran, highlighting regional variations in tick vector competency and their role in the epidemiology of ovine babesiosis [23]. In this study, we aimed to investigate the natural transovarial transmission of *B. ovis* in engorged female *R. bursa* and *R. turanicus* ticks collected from small ruminants, as well as in their unfed larvae.

## 2. Materials and Methods

### 2.1. Tick Collection and Identification

This work was carried out between May and June 2020 in Elazığ Province of Türkiye. Apparently healthy sheep (*n* = 41) and goats (*n* = 43) brought to a local abattoir from the surroundings of Elazığ Province were examined for ixodid tick infestations. All visible ticks were carefully removed from the animals and placed into labeled tubes. Following morphological identification under a stereomicroscope using standard taxonomic keys, adult ticks were classified to the species level [24]. Male ticks were utilized to assist in the species‐level identification of the engorged females. Among them, fully engorged and suitable‐for‐oviposition females of *R. bursa* and *R. turanicus* were selected and included in the study.

The selected ticks were placed individually into sterile plastic vials with perforated caps and maintained in a controlled incubator at 27^°^C ± 1^°^C, 80%–85% relative humidity (RH) to facilitate oviposition. Incubation conditions were monitored daily using a hygrometer and thermometer to ensure consistency. After the completion of oviposition, female tick carcasses were removed, surface sterilized with 70% ethanol, and bisected using scalpels. They were stored at −20°C in a freezer for subsequent genomic DNA extraction. Egg masses were kept in the same incubator conditions (27^°^C ± 1^°^C, 80%–85% RH) until larval hatching occurred. After hatching was completed, approximately 100 larvae were collected from each engorged female tick and pooled. To enable subsequent molecular analyses, larval pools were also stored at −20°C until DNA extraction.

### 2.2. DNA Extraction and Molecular Analyses

Each larval pool and adult female carcass was individually homogenized in 2 mL safe‐lock microcentrifuge tubes containing bead‐beating using a TissueLyser II (Qiagen, Hilden, Germany), in accordance with the manufacturer′s protocol. Total genomic DNA was isolated using the PureLink Genomic DNA Mini Kit (Invitrogen, Carlsbad, CA, United States) following the standard protocol recommended by the supplier. After extraction, DNA samples were immediately transferred to sterile microcentrifuge tubes and stored at 4°C until they were processed for molecular analyses.

The isolated DNA was utilized as a template in nested PCR assays to detect the *18S rRNA* gene of *B. ovis* using two primer sets and the protocol previously reported in the literatures [25, 26]. For the amplification of *B. ovis*, the first PCR was performed using the Nbab1F/Nbab1R primers, followed by a second‐round PCR using the Bbo‐F/Bbo‐R primers. Before performing PCR for *B. ovis*, DNA samples isolated from female tick carcasses and larval pools were subjected to amplification targeting the tick‐specific *16S rDNA* gene [27]. The positive samples were subsequently analyzed for the presence of *B. ovis*. The primers (primer sequence 5 ^′^‐3 ^′^, product size [base pair]) used in this study are presented in Table 1.

**Table 1:** The primer (primer sequence 5 ^′^‐3 ^′^, amplicon size) applied in this study.

**Target organism**	**Target gene**	**Primer (F/R)**	**Amplicon size**	**Reference**
*Babesia/Theileria* spp.	*18S rRNA*	Nbab1F/Nbab1R	1600 bp	[26]
*B. ovis*	*18S rRNA*	Bbo‐F/Bbo‐R	549 bp	[27]
Ixodid tick	*16S rDNA*	16S+1/16S‐1	460 bp	[28]

PCR amplifications were performed using a thermal cycler (Labcycler Gradient, Göttingen, Germany). Ten microliters of each PCR product was separated by electrophoresis on a 1.3% agarose gel for 30 min and then visualized with a Quantum Vilber Lourmat gel documentation system (Marne‐la‐Vallée, France). Each PCR reaction included both positive and negative controls: DNA from *B. ovis*‐infected *R. bursa* (female carcass of *R. bursa* infected with *B. ovis* derived from the laboratory colony infected with *B. ovis*) was used as positive control. *B. ovis*‐free *R. bursa* derived from the sterile laboratory colony was also used as negative control [28].

## 3. Results

### 3.1. Identification of the Collected Ticks and Harvesting of Larval Pools

In this survey, a total of 751 adult ixodid ticks, comprising 271 females and 480 males, were collected from 84 small ruminants, including 41 sheep and 43 goats. Morphological examination of the specimens revealed three tick species: *R. bursa* (*n* = 353; 134 females and 219 males), *R. turanicus* (*n* = 355; 129 females and 226 males), and *Hyalomma marginatum* (*n* = 43; eight females and 35 males) (Table 2).

**Table 2:** Number of collected ticks from sheep and goats.

**Host**	**Collected tick species**					
	***R. bursa***		***R. turanicus***		***H. marginatum***	
	**Female**	**Male**	**Female**	**Male**	**Female**	**Male**
Sheep	98	142	64	119	7	21
Goat	36	77	65	107	1	14
Total	134	219	129	226	8	35

Among them, 85 fully engorged females and suitable for oviposition were incubated to allow them to lay eggs. These included 45 females from *R. bursa* and 40 from *R. turanicus*. Following incubation, 80 out of the 85 engorged females successfully oviposited, while the remaining five did not lay eggs. Larval emergence was observed from all egg batches (Table 3).

**Table 3:** Detection of *B. ovis* in fully engorged female *R. bursa* and *R. turanicus* carcasses and their larval pools.

**Tick species**	**No. of total female ticks incubated**	**No. of egg-laying female carcasses**	**No. of larval pools** ^**a**^ **(no. of total larvae examined)**	**No. of positive carcasses**	**No. of positive larval pools**
*R. bursa*	45	42	42 (4200)	8/42 (19.04%)	8/42 (19.04%)
*R. turanicus*	40	38	38 (3800)	3/38 (7.89%)	0/38
Total	85	80	80 (8000)	11/80 (13.75%)	

^a^Each larvae pool included 100 larvae.

### 3.2. Detection of *B. ovis* in Engorged Female *R. bursa* and *R. turanicus*, as Well as in Their Larval Pools

**Figure 1:**
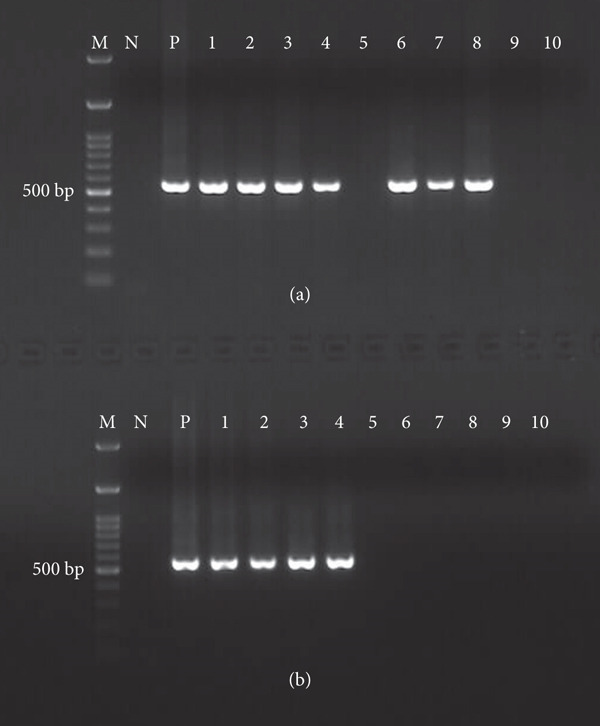
Representing gel imaging of nested PCR results of female carcasses and their larval pools of *R. bursa* and *R. turanicus*. (a) Gel image showing positive and negative PCR amplification products of *R. bursa* and *R. turanicus* carcasses. M: 100 bp marker. N: negative control (*B. ovis*‐free *R. bursa* derived from the sterile laboratory colony). P: positive control (female carcass of *R. bursa* infected with *B. ovis* derived from the laboratory colony infected with *B. ovis*). Lanes 1−4 show positive PCR amplification of female *R. bursa* carcasses collected from naturally infected with *B. ovis*. Lane 5 shows negative amplification of representing female *R. bursa* carcass. Lanes 6–8 indicate positive PCR amplification from the female *R. turanicus* carcasses collected from sheep naturally infected with *B. ovis*. Lanes 9 and 10 show negative amplification of representing female *R. turanicus* carcasses. (b) Gel image showing representing positive and negative PCR amplification products of larval pools derived from PCR positive and negative *R. bursa* and *R. turanicus* carcasses. Lanes 1−4 indicate positive larval pools derived from PCR positive female *R. bursa* carcasses. Lane 5 shows PCR negative larvae pool derived from PCR negative female *R. bursa* carcass. Lanes 6−8 show negative larval pools derived from PCR positive female *R. turanicus* carcasses. Lanes 9 and 10 show negative larval pools derived from PCR negative female *R. turanicus* carcasses.

According to the molecular analysis results, *B. ovis* DNA was detected in 19.04% (8/42) and 7.89% (3/38) of the engorged female carcasses of *R. bursa* and *R. turanicus*, respectively. Notably, *B. ovis* amplification was observed in all larval pools derived from PCR‐positive *R. bursa* females. In contrast, no positive amplification product was obtained from any of the larval pools derived from PCR‐positive *R. turanicus* (Table 3 and Figure 1).

## 4. Discussion

The term “vector competence of ixodid ticks” refers to the ability to acquire and develop the pathogen and transmit it to a susceptible new vertebrate host. This dual mode of transmission enhances the persistence of the pathogen in both tick populations and mammalian hosts, contributing to the maintenance of the disease cycle in endemic areas [29, 30]. On the other hand, vector capacity includes environmental factors such as humidity, temperature, tick density, and host preferences and expresses the potential to transmit pathogens in a specific region at a specific time [29, 30]. Understanding the vector competence, capacity, and transmission dynamics of tick species is therefore essential for evaluating their epidemiological role and implementing effective control strategies [29, 31, 32]. Ixodid tick species are capable of successfully transmitting *Babesia* species acquired during blood feeding on suitable vertebrate hosts in their adult stage to the next generation, and they are considered not only vectors but also reservoirs [1, 4]. In general, tick species capable of transovarial transmission are recognized as significant vectors for the pathogens they carry. However, this assumption should be supported not only by experimental evidence but also by field‐based data, as laboratory findings may not always reflect natural ecological dynamics. A tick species may experimentally transmit a specific pathogen through transstadial and/or transovarial routes [33]. However, due to biological or ecological constraints—such as host specificity or feeding preferences—it may play no role in the natural transmission cycle of the pathogen [4]. For instance, it has been demonstrated that *H. marginatum* can transmit *Theileria annulata* under laboratory conditions [33]. However, this tick is not considered a natural vector for the parasite because its immature stages rarely feed on cattle, the main reservoir host of the pathogen [4]. This highlights the importance of integrating field surveys into the study of vector–pathogen dynamics. Furthermore, natural transmission studies provide crucial insights into the ecoepidemiology of tick‐borne protozoan infections, particularly for agents such as *Babesia* and *Theileria*. *B. ovis* is among the first protozoan parasites to be identified as being transmitted by vector ticks [31]. It has been reported that species in the *Babesia* sensu stricto clade (Clade X) can be transmitted by ixodid ticks through both transstadial and transovarial routes [8, 31]. In the present study, a field‐based investigation was conducted to determine whether *R. bursa* and *R. turanicus* play a role in the natural transmission of *B. ovis*, the etiological agent of ovine babesiosis. Our findings revealed that *B. ovis* DNA was detected in eight out of 42 (19.04%) fully engorged female *R. bursa* ticks collected from naturally infested sheep and goats. Notably, the larval pools originating from these infected females also tested positive for *B. ovis*, providing strong molecular evidence for transovarial transmission of the parasite. This indicates that *R. bursa* not only acquires *B. ovis* from infected hosts during blood feeding but is also capable of vertical transmission to its progeny. These findings constitute strong evidence of vector competence in *R. bursa* for the transmission of *B. ovis* under natural conditions, consistent with previous experimental studies [14, 21, 22] and field‐based reports [7, 17–20, 23, 25, 34]. Furthermore, the ability of *R. bursa* to maintain the parasite across generations implies a possible role as a reservoir, contributing to the persistence of *B. ovis* in endemic areas.

In previous studies, *R. turanicus* has been implicated as a potential vector in the transmission of *B. ovis* [23, 35–37]. In our study, no parasite DNA was detected in the larval pools obtained from the fully engorged female ticks that tested positive for *B. ovis* by PCR, indicating no evidence of vertical (transovarial) transmission. These results suggest that although *R. turanicus* may ingest the parasite during blood feeding on infected hosts, it is unlikely to sustain and transmit *B. ovis* to its offspring. This implies that *R. turanicus* may not serve as a competent biological vector for *B. ovis*. However, to confirm the vectorial capacity of *R. turanicus* for the parasite, controlled experimental transmission studies should be done. Additionally, ecological and seasonal factors influencing tick–pathogen dynamics should be considered in future investigations to better define the role of this species in the epidemiology of ovine babesiosis caused by *B. ovis*.

## 5. Conclusion

This study provides field‐based molecular evidence supporting the role of *R. bursa* as a competent biological vector for *B. ovis*. The detection of *B. ovis* DNA in both engorged females and their larval progeny strongly indicates the occurrence of transovarial transmission under natural conditions. These findings, consistent with previous experimental and field studies, further suggest that *R. bursa* may function not only as a vector but also as a reservoir, facilitating the persistence of the parasite in endemic areas. In contrast, while *R. turanicus* females collected from naturally infected hosts tested positive for *B. ovis*, the absence of parasite DNA in their larval progeny suggests a lack of vertical transmission and raises questions about their role as a competent vector. Although *R. turanicus* may acquire the pathogen during blood feeding, current data do not support its ability to transmit *B. ovis* to subsequent generations. Therefore, further experimental and ecological studies are needed to clarify its role in the parasite′s transmission cycle.

## Conflicts of Interest

The authors declare no conflict of interest.

## Funding

This study was funded by the Türkiye Bilimsel ve Teknolojik Araştırma Kurumu (10.13039/501100004410) (221O119).

## Data Availability

The data that support the findings of this study are available upon request from the corresponding author. The data are not publicly available due to privacy or ethical restrictions.
